# Risk and predictors for early radiation pneumonitis in patients with stage III non-small cell lung cancer treated with concurrent or sequential chemoradiotherapy

**DOI:** 10.1186/1748-717X-9-172

**Published:** 2014-07-30

**Authors:** Jun Dang, Guang Li, Shuang Zang, Shuo Zhang, Lei Yao

**Affiliations:** 1 Department of Radiation Oncology, The First Hospital of China Medical University, No.155 Nanjing Road, Heping District, Shenyang 110001, China; 2 Department of Nursing, China Medical University, Shenyang 110001, China

**Keywords:** Non-small cell lung cancer, Radiation pneumonitis, Predictor, Concurrent chemoradiotherapy, Sequential chemoradiotherapy

## Abstract

**Background:**

The rate of radiation pneumonitis (RP) for patients receiving chemoradiotherapy has been various across studies. Whether it is related to different chemotherapy schedules used in combination with radiation therapy were evaluated in this study. New factors associated with RP were also investigated.

**Methods and materials:**

A total of 369 consecutive patients with Stage III non small cell lung cancer treated with chemoradiotherapy were followed after radiotherapy (RT). Among them 262 patients received concurrent chemoradiotherapy followed by consolidation chemotherapy and 107 patients received only sequential chemotherapy after RT. RP was graded according to Common Terminology Criteria for Adverse Events version 4.0.

**Results:**

The rate of grade ≥ 2 were 39.7%, 31% and 33.6% in the concurrent DP (docetaxel/cisplatin), concurrent NP (vinorelbine/cisplatin) and sequential group, and grade ≥ 3 RP were 18.4%, 9.5%, and 11.2% respectively. The rate of grade ≥ 3 RP was significantly higher in concurrent DP group than that in concurrent NP group (p = 0.04). RP occurred earlier in concurrent DP group than that in the other two groups. There were no significant differences in response rate among the three groups. In the multivariate analysis, age (OR = 1.99, p = 0.038 and OR = 8.90, p < 0.001), chemotherapy schedule (OR = 1.45, p = 0.041 and OR = 1.98, p = 0.013), mean lung dose(OR = 1.42, p < 0.001 and OR = 1.64, p < 0.001), and planning target volume(OR = 1.004, p = 0.001 and OR = 1.005, p = 0.021) were predictors for both grade ≥ 2 and grade ≥ 3 RP. Response to treatment was a new predictor for grade ≥ 3 RP only (OR = 4.39, p = 0.034).

**Conclusions:**

Response to treatment was found to be a new predictor for grade ≥ 3 RP. Compared to concurrent NP schedule, concurrent DP schedule achieved similar response to treatment but resulted in a higher risk of grade ≥ 3 RP.

## Background

Approximately 30% of patients with non small cell lung cancer (NSCLC) are diagnosed at a locally advanced stage. In unresectable locally advanced lung cancer (LA-NSCLC), compared to radiation therapy (RT) alone, the addition of cisplatin based sequential chemotherapy to radiation therapy resulted in modest improvement in survival [1,2]. Concurrent cisplatin based chemoradiotherapy showed consistent improvement in survival compared with sequential chemoradiotherapy [3-5]. Concurrent chemoradiotherapy (CCRT) has been regarded as the standard treatment for unresectable LA-NSCLC patients with good performance status.

Treatment-related acute pulmonary toxicity for patients receiving chemoradiotherapy has been various across studies (4.8% to 47%), and various chemotherapy drugs (e.g., taxanes, pemetrexed, irinotecan, gemcitabine) were used (Table 1). Whether the differences in risk of radiation pneumonitis (RP) are related to different chemotherapy drugs or chemotherapy schedules used in combination with RT need further investigated.

**Table 1:** Results of RP risk in clinical studies for CCRT

**Study**	**Patients (n)**	**Treatment schedule**	**RT dose (Gy)**	**RP**
Belani et al. [6]	74	PC + RT followed by PC	63	Grade ≥3 16%
Gandara et al. [7]	83	PE + RT followed by docetaxel	61	Grade ≥3 7%
Fournel P et al. [3]	100	PE + RT followed by NP	60	Grade ≥2 5%
Xu Y et al. [8]	21	pemetrexed/carboplatin + RT followed by pemetrexed/carboplatin	NR	Grade ≥3 4.8%
Bastos BR et al. [9]	32	carboplatin/irinotecan + RT followed by docetaxel	63	Grade ≥2 42%
Eroglu C et al. [10]	93	DP + RT followed by DP	66	Grade ≥3 9%
Phernambucq EC et al. [11]	83	cisplatin/gemcitabine + RT followed by PE	44-66	Grade ≥3 7.9%
Wang S et al. [12]	223	PC + RT followed by PE	63	Grade ≥3 32%
Onishi H et al. [13]	32	weekly docetaxel	60-66	Grade ≥3 47%

*RP*, radiation pneumonitis; *CCRT*, concurrent chemoradiotherapy; *RT*, radiotherapy; *PC*, carboplatin/paclitaxel; *PE*, cisplatin/etoposide; *DP*, docetaxel/cisplatin.

In this study, we evaluated the risk of RP in patients with Stage III NSCLC treated with concurrent or sequential chemoradiotherapy respectively. We also analyzed clinical and dose-volume factors correlated with the development of RP.

## Methods and materials

### Patients

From October 2009 through August 2013, 427 consecutive patients with stage III NSCLC received concurrent or sequential chemoradiotherapy at the Radiotherapy Department of The First Hospital of China Medical University. These patients were recruited into our study. Patients were staged according to the new staging system initiated by the International Association for the Study of Lung Cancer in 2009 [14]. Patients were excluded if they were treated with inconsistent doses per fraction (n = 12), treated with inconsistent chemotherapy schedules or drugs (n = 28), or a total radiation dose less than 50 Gy (n = 17). Thus, 369 patients were followed up prospectively after RT was completed.

### Radiation therapy

All of the patients underwent three-dimensional conformal radiotherapy (3D-CRT) or intensity-modulated radiotherapy (IMRT). Treatments planning computed tomography (CT) scans with slices 5 mm thick were obtained from the mandible to the lower edge of the liver before RT. The gross tumor volume (GTV) included the primary disease as well as any involved regional lymph nodes, which were defined as those with a short-axis diameter of at least 1 cm on CT scan or with a short-axis diameter of less than 1 cm but with high fluorodeoxy-glucose (FDG) uptake on PET-CT scan. The clinical target volume (CTV) was defined as the GTV plus a 0.6 cm- 0.8 cm margin. The planning target volume (PTV) was the CTV with 0.5–1.0 cm margin. The prescribed dose was 60–66 Gy in 2.0-2.2 Gy daily fractions. Five fractions a week were usually used.

### Chemotherapy

262 patients received concurrent chemoradiotherapy, and 107 patients received sequential chemoradiotherapy. There were two chemotherapy regimens (DP and NP) for the concurrent group. Concurrent DP regimen consisted of 20–25 mg/m^2^/d of cisplatin on days 1–3 and 65–70 mg/m^2^/d of docetaxel on day 1; Concurrent NP regimen consisted of 20–25 mg/m^2^/d of cisplatin on days 1–3 and 20 mg/m^2^/d of vinorelbine on days 1 and 5. Chemotherapy was initiated simultaneously with RT, 1 to 2 cycles was concurrently administered with radiotherapy and 2 to 3 cycles was administered within 4 weeks of completing RT (for 28% of patients in concurrent NP group, NP regimen was concurrently administered with radiotherapy, but DP regimen was administered in followed consolidation chemotherapy). The chemotherapy regimen for sequential group was DP (20–25 mg/m^2^/d of cisplatin on days 1–3 and 70–75 mg/m^2^/d of docetaxel on day 1); chemotherapy was initiated 2 to 4 weeks after RT, 3 to 4 cycles was administered, and every 3–4 week a cycle.

### Dose-volume histogram (DVH) parameters

The total normal lung volume was defined as the total lung volume minus the primary CTV and the volume of the trachea and main bronchi. The following parameters were extracted for modeling: V20 (total lung volume receiving ≥20 Gy), mean lung dose (MLD), mean heart dose (MHD) and planning target volume (PTV).

### Evaluation and follow-up

Early RP and late lung fibrosis are different stages of radiation-induced lung injury. Early RP usually occurs 1 to 6 months after RT, whereas late lung fibrosis usually occurs 6–24 months after RT. Because we were interested in early RP, we used six months as the cut-off for diagnosis.

Patients were evaluated by radiation oncologists weekly during radiation, and once a month until six months after RT. A chest CT scan was performed at each follow-up evaluation after completion of radiotherapy. A diagnosis of RP was made with consensus by at least two radiation oncologists based on clinical symptoms, with or without radiographic infiltrate changes. Findings on a CT image of RP include a diffuse haziness or fuzziness in the areas of the irradiated lung, which may coalesce to a form a relatively sharp edge corresponding to the shape and size of the radiation field. These radiographic changes in RP may also reveal outside the radiation field. The symptoms of RP are dry cough, low-grade fever, chest pain, and shortness of breath. Cases difficult to diagnose were referred to respiratory or circulation physicians to exclude other diseases. Patients with a diagnosis of grade ≥ 2 RP were required to have an immediate intervention, including oral or intravenous steroids, oxygen, and antibiotics. Grading was conducted according to Common Terminology Criteria for Adverse Events version 4.0 [15].

Response Evaluation Criteria in Solid Tumors (RECIST) was used to evaluate treatment response 4–6 weeks after the completion of the treatments.

### Statistical analysis

Grade2 or above RP were counted as events. Statistical analysis was performed using SPSS 13.0 statistical software (Chicago, IL). Univariate analysis was performed to evaluate the influence of patient characteristics and dose-volume variables on RP risk. The independent samples *t*-test, analysis of variance or the *χ*^2^-test were used for univariate analysis. Multivariate analysis was performed using logistic regression model (enter method) containing all variables that attained or trended toward univariate statistical significance (p ≤ 0.3) in Table 2. The receiver operating characteristic (ROC) curve was used to identify the c-statistics for each dose-volume variable and the models. The correlation between dose-volume variables was tested with Pearson Correlation Coefficient. All statistical tests were 2-sided and p ≤ 0.05 was considered statistically significant.

**Table 2:** Clinical and treatment characteristics (n = 369)

**Characteristics**	**No. of patients**
Sex (male/female)	263(71%)/106(29%)
Age (≥70/<70)	117(32%)/252(68%)
Performance status (0/1/2)	114(31%)/244(66%)/11(3%)
Stage (IIIA/IIIB)	234(63%)/135(37%)
Pathology (squamous/adenocarcinoma/other)	229(62%)/119(32%)/21(6%)
CTV location	
Left/right	193(52%)/176(48%)
Upper/lower	235(64 %)/134(36%)
Smoking history (yes/no)	230(62%)/139(38%)
Response (PR + CR/others)	280(76%)/89(24%)
RT (3D-CRT/IMRT)	288(78%)/81(22%)
Chemotherapy schedule (concurrent DP/concurrent NP/sequential)	136(37%)/126(34%)/107(29%)

*CTV*, clinical target volume; *CR*, complete response; *PR*, partial response; *DP*, docetaxel/cisplatin; *NP*, vinorelbine/cisplatin; *3D-CRT*, three-dimensional conformal radiotherapy; *IMRT*, intensity-modulated radiotherapy.

## Results

Four patients died of grade 5 RP (three in concurrent DP group, and one in concurrent NP group). All other patients were successfully followed up through the end point of six months after RT.

### Response to treatment

The response rates (CR + PR) were 78.7% in concurrent DP, 77% in concurrent NP, and 71% in sequential group respectively, which were not significantly different (P = 0.585).

### Evaluation of RP

RP occurred earlier in concurrent DP group (2.4 ± 1.1 months after RT) than that in concurrent NP (2.9 ± 1.2 months after RT) and sequential group (3.2 ± 1.2 months after RT) (p < 0.05 for each comparison).

The rate of grade ≥ 2 were 39.7%, 31% and 33.6% in the concurrent DP, concurrent NP and sequential group, and grade ≥ 3 RP were 18.4%, 9.5%, and 11.2% respectively. The rate of grade ≥ 3 RP was significantly higher in concurrent DP group than that in concurrent NP group (p = 0.04).

#### ***Patient characteristics***

Patient characteristics were listed in Table 2. The results of univariate analysis were shown in Table 3. The results of the multivariate analysis suggested that age and chemotherapy schedule were predictors for both grade ≥ 2 (OR = 1.99, p = 0.038; OR = 1.45, p = 0.041) and grade ≥ 3 RP (OR = 8.90, p = 0.000; OR = 1.98, p = 0.013). Response to treatment was a predictor for grade ≥ 3 RP only (OR = 4.39, p = 0.034) (Table 4).

**Table 3:** Demographic and tumor characteristics and dose-volume factors of patients by RP status

**Variables**	**Grade 0-1**	**Grade 2**	**Grade ≥ 3**	**p-value**
	**n = 240**	**n = 80**	**n = 49**	
Sex (male/female)	174/66	57/23	32/17	0.598
Age (≥70/<70)	72/168	22/58	23/26	0.044
Performance status (0/1/2)	74/157/9	24/55/1	16/32/1	0.808
Stage (IIIA/IIIB)	159/81	46/34	29/20	0.299
Pathology (squamous/adenocarcinoma/others)	145/81/14	54/23/3	30/15/4	0.723
CTV location				
Left/right	115/123	48/32	30/19	0.083
Upper/lower	164/76	48/32	23/26	0.013
Smoking history (Y/N)	153/87	49/31	28/21	0.668
Response (PR + CR/others)	173/67	62/18	45/4	0.012
RT (3D-CRT/IMRT)	189/51	63/17	36/13	0.708
Chemotherapy schedule (concurrent DP/concurrent NP/sequential)	82/87/71	29/27/24	25/12/12	0.268
PTV	396.67 ± 107.18	460.59 ± 105.02	514.92 ± 98.47	0.000
V20	0.28 ± 0.09	0.37 ± 0.09	0.43 ± 0.10	0.000
MLD	14.15 ± 3.26	17.71 ± 3.28	21.40 ± 3.48	0.000
MHD	15.24 ± 7.16	20.33 ± 6.36	22.31 ± 5.62	0.000

*RP*, radiation pneumonitis; *CTV*, clinical target volume; *PTV*, planning target volume; *V20*, lung volume receiving ≥20Gy; *MLD*, mean lung dose; *MHD*, mean heart dose; *CR*, complete response; *PR*, partial response; *DP*, docetaxel/cisplatin; *NP*, vinorelbine/cisplatin; *3D-CRT*, three-dimensional conformal radiotherapy; *IMRT*, intensity-modulated radiotherapy.

**Table 4:** C-statistics for dose-volume variables

**Variables**	**Grade ≥ 2**		**Grade ≥ 3**	
	**c-statistics**	**95% CI**	**c-statistics**	**95% CI**
V20	0.808	0.761 ~ 0.855	0.827	0.771-0.883
MLD	0.838	0.794 ~ 0.881	0.897	0.854-0.939
MHD	0.727	0.674-0.779	0.725	0.655-0.796
PTV	0.720	0.667-0.774	0.757	0.692-0.822

*PTV*, planning target volume; *V20*, lung volume receiving ≥20 Gy; *MLD*, mean lung dose; *MHD*, mean heart dose.

#### ***Dose-volume factors***

In univariate analysis, the dose-volume variables of V20, MLD, MHD and PTV were all significantly associated with RP (p = 0.000 for each comparison) (Table 3). C-statistics for each dose-volume variable were shown in Table 5. Due to the linear correlation between MLD and V20 (r = 0.851, p = 0.000), and MLD had the higher c-statistic than V20 (grade ≥ 2: 0.838 vs. 0.808; grade ≥ 3: 0.897 vs. 0.827), V20 was removed from the logistic regression model. MLD, MHD and PTV were included in the multivariate logistic regression analysis. The results of the multivariate analysis suggested that MLD and PTV were predictors for both grade ≥ 2 (OR = 1.42, p < 0.001 and OR = 1.004, p = 0.001) and grade ≥ 3 RP (OR = 1.64, p < 0.001 and OR = 1.005, p = 0.021) (Table 4). The c-statistic was 0.86 for the Grade ≥ 2 model and 0.94 for the Grade ≥ 3 model, indicating good discrimination.

**Table 5:** Multivariate logistic regression analysis of factors associated with RP

**Variables**	**Grade ≥ 2**			**Grade ≥ 3**		
	**OR**	**95% CI**	**p-value**	**OR**	**95% CI**	**p-value**
Age (≥70/<70)	1.99	1.04-3.80	0.038	8.90	3.23-24.51	<0.001
Chemotherapy schedule (concurrent DP/concurrent NP/sequential)	1.45	1.01-2.07	0.041	1.98	1.16-3.39	0.013
Response (PR + CR/others)	1.06	0.53-2.14	0.870	4.39	1.12-17.24	0.034
PTV	1.004	1.002-1.007	0.001	1.005	1.001-1.009	0.021
MLD	1.42	1.28-1.58	<0.001	1.64	1.40-1.93	<0.001
Stage (IIIA/IIIB)	1.28	0.72-2.26	0.405	0.85	0.36-2.01	0.705
CTV location (left/right)	0.75	0.43-1.32	0.32	1.43	0.60-3.44	0.419
CTV location (upper/lower)	0.68	0.33-1.40	0.29	0.74	0.25-2.18	0.581

*RP*, radiation pneumonitis; *PTV*, planning target volume; *MLD*, mean lung dose; CR, complete response; *PR*, partial response; *DP*, docetaxel/cisplatin; *NP*, vinorelbine/cisplatin; *CTV*, clinical target volume.

## Discussion

Combination of chemotherapy and RT has been well reported increasing the risk of pulmonary injury, either sequential [16] or concurrent [12,13]. Weekly docetaxel with concurrent conventional radiotherapy resulted in 47% grade ≥3 RP [13]; Carboplatin plus taxanes with concurrent 3D-CRT resulted in 32% grade ≥3 RP [12]. However, there are also inconsistent results. PE regimen showed 5% grade ≥2 RP [3], and pemetrexed plus carboplatin regimen showing 4.8% grade ≥3 RP [8] with concurrent 3D-CRT in two Phase III trials. Data from a large meta-analysis of predictors of RP showed that concurrent carboplatin/paclitaxel regimen was associated with a high risk of RP compared with concurrent cisplatin/etoposide regimen [17]. In this study, we compared the risk of RP among concurrent DP, concurrent NP and sequential group respectively. Our data showed a higher rate of grade ≥ 3 RP for concurrent DP schedule (18.4%) than that for concurrent NP schedule (9.5%)(p < 0.05), while the risk of RP for sequential schedule was moderate (11.2% for grade ≥ 3). Chemotherapy schedule was an independent predictor for grade ≥ 3 RP (OR = 1.98, p = 0.013). The results above suggested that differences in RP risk across studies might be related to different chemotherapy schedule or drugs used in chemoradiotherapy. Thus care should be taken when chemotherapy schedule or drugs are selected in combination with RT to ensure that treatment toxicities do not overwhelm the potential benefits of treatment.

There were no significant differences in the response rate among concurrent DP (78.7%), concurrent NP (77%) and sequential group (71%) in this study (P = 0.585). However, it was interesting to note a significantly higher rate of RP for patients with good response to treatment (CR or PR) than others (p < 0.05) (Table 3), and the response to treatment was an independent predictor for grade ≥3 RP in Multivariate analysis(OR = 4.39, p = 0.034) (Table 4). To our knowledge, no study has been reported to analyze the correlation between the response to treatment and RP. How to explain the result? Most of our patients were with relatively large tumor size(T3), and treated with concurrent DP or concurrent NP RT. Chemotherapy drugs thought to be radiosensitization might lead to rapid response to treatment during process of RT. If treatment planning were not modified in time, the original GTV would contain part of lung leading to higher dose to larger volume of lung. If so, modifying treatment planning in time during process of RT for patients receiving concurrent chemoradiotherapy would become definitely necessary.

MLD and age were predictors for RP in this study, which were consistent with the literature. Results from studies about the correlation between PTV and the risk of RP remain inconsistent [18,19]. It might be related to differences in total lung volume of different patients; that is, a lower percentage of lung would be irradiated in patients with a larger lung volume and a higher percent of lung would be irradiated in patients with a smaller lung volume when their PTV were equal. PTV was a predictor for both grade ≥ 2 and grade ≥ 3 RP in our study, however, the correlation was weak (OR = 1.004 and OR = 1.005, respectively) (Table 4).

Rodrigues G, et al. [20] performed a systematic review of the predictive ability of various dose–volume variables (V_dose_, MLD) for RP and found that most studies did show an association between dose–volume variables and RP risk, however, the predictive ability was generally poor. In this study, dose–volume variables such as MLD and V20 had the higher c-statistics for Grade ≥ 2 or Grade ≥ 3 RP, indicating good discrimination (Table 5).

In this study, RP occurred earlier in concurrent DP group than that in concurrent NP or sequential group (*p* < 0.05). It was probably due to damage to lung by docetaxel used in concurrent RT process.

## Conclusions

Compared to concurrent NP schedule, concurrent DP schedule achieved similar response to treatment but resulted in a higher risk of RP. Besides age, PTV, and MLD, response to treatment and chemotherapy schedule were found to be new predictors for RP.

### Consent

Written informed consent was obtained from the patient for the publication of this report and any accompanying images.

## Competing interests

The authors declare that they have no competing interests.

## Authors’ contributions

JD and GL are lead authors who participated in manuscript drafting, table creation, and manuscript revision. SZ performed statistical analyses. SZ participated in the clinical coordination and aided in data collection. LY is the dosimetrist who contributed dosimetric data and tables. All authors read and approved the final manuscript.
